# Are childhood factors predictive of adult health literacy? A longitudinal birth cohort analysis

**DOI:** 10.1016/j.ssmph.2023.101426

**Published:** 2023-05-08

**Authors:** I. Solis-Trapala, P. Campbell, R.J. Lacey, G. Rowlands, K.M. Dunn, J. Protheroe

**Affiliations:** a School of Medicine, Keele University, Staffordshire, ST5 5BG, UK; b Midlands Partnership NHS Foundation Trust, St Georges' Hospital, Stafford, ST16 3AG, UK; c Population Health Sciences Institute, Newcastle University, Newcastle Upon Tyne, NE1 7RU, UK

**Keywords:** Health literacy, ALSPAC, Children, Education, Mental health, Life course, Birth cohort, Maternal health

## Abstract

Health literacy (HL), defined as the ability of an individual to understand and appraise health information to make informed decisions on their health, helps maintain and improve one's health and thus reduce the use of healthcare services. There is a recognised global effort to address insufficient HL in early life and understand how HL develops. This study examined the association of a range of factors including educational, speech and language ability, health and healthcare engagement, sleep problems, mental health, demographic, environmental, and maternal factors at different childhood stages (from 5 years to 11 years) with later adult HL at age 25. HL was measured using a HL ordinal score (insufficient, limited, or sufficient) derived from the European Literacy Survey Questionnaire-short version (HLS-EU-Q16) within a large UK based birth cohort (Avon Longitudinal Study of Parents and Children: ALSPAC study). Univariate proportional odds logistic regression models for the probability of having higher levels of HL were developed.

Results of analysis of 4248 participants showed that poorer speech and language ability (aged 9 years, OR 0.18 95% CI 0.04 to 0.78), internalising in child (age 11 years, OR 0.62 95% CI 0.5 to 0.78), child depression (age 9 years, OR 0.67 95% CI 0.52 to 0.86), and the presence of maternal depression (child age 5, OR 0.80 95% CI 0.66 to 0.96), reduced the odds of sufficient HL when adult. Our results suggest some useful markers to identify children at potential risk of low HL that could be targeted for research into future interventions within school settings, for example, child's speech and language capability. In addition, this study identified child and maternal mental health as factors associated with later development of limited HL and future research should consider what potential mechanisms might explain this link.

## Highlights

• Health literacy is an important determinant of an individual's health.
• Greater understanding is needed on how health literacy develops in childhood.
• Birth cohort data was used to identify factors associated with adult health literacy.
• Speech and language ability, child and maternal mental health are key factors.

## 1 Introduction

Health literacy (HL) is described as the measure of “*people's knowledge, motivation and competencies to access, understand, appraise, and apply health information in order to make judgments and take decisions in everyday life concerning healthcare, disease prevention and health promotion to maintain or improve quality of life during the life course*” (Sørensen et al., 2012). Lower levels of health literacy are more likely found within adults who have limited levels of education attainment and experience higher levels of deprivation (HLS-EU Consortium, 2012). Having low health literacy is linked to significant health issues among adults, such as poorer overall health, less effective management of health conditions, less use of preventative care, increased use of health services, and, among the elderly, increased mortality (Berkman et al., 2011; Kickbusch et al., 2013). The World Health Organisation (WHO) has recognised the global need to strengthen health literacy to address inequity (World Health Organization, 2016). Importantly, the WHO points out that intervention to improve health literacy in early life offers an optimum opportunity to influence the “life chances of children” and confer health benefits that could extend into adulthood (World Health Organisation. Regional Office for Europe et al., 2016). Key to understanding health literacy within child/adolescent populations involves not only direct assessment of the child's health literacy ability, which has presently proved challenging due to current discord on how to assess child health literacy that matches the constant shift in child cognitive developmental stages (Guo et al., 2018; Okan et al., 2018), but is also importantly consideration of wider determinants such as parental/caregiver influence, differential illnesses associated with childhood/adolescence, and demographic factors (Forrest et al., 1997; Guo et al., 2018). Previous research has demonstrated a tentative link between child/adolescent educational ability and later adult health literacy and general literacy (Clouston et al., 2017; Richards et al., 2009), but, to our knowledge very little study to date has considered what components of educational ability are important (e.g. Maths, Science, English, Speech and Language), and what role potential wider determinants may have such as the child's physical and emotional health, previous engagement with healthcare, and family and environmental influences. This information is important as it can inform on which factors within childhood are predictive of later health literacy to help understand how health literacy might develop, but also to potentially inform the design of appropriate and effective early life interventions to improve health literacy (Bröder et al., 2017). This current study offers a unique life-course perspective by using prospectively collected data from a large birth cohort to examine factors within childhood that are associated with later adult health literacy.

## 2 Methods

### 2.1 Design and population

This study utilises data gathered from the Avon Longitudinal Study of Parents and Children (ALSPAC) study (Boyd et al., 2013; Fraser et al., 2013). ALSPAC is a longitudinal birth cohort which enrolled 14,541 pregnant women who resided in one of three Bristol-based health districts in England with expected delivery date between April 1, 1991 and December 31, 1992. When the oldest children were approximately 7 years old, the initial sample was bolstered with eligible cases who had failed to join the study originally. Therefore, there is data available for more than the 14,541 pregnancies mentioned above (Northstone et al., 2019). These children, their mothers and partners have been followed up regularly over the past three decades using a combination of postal questionnaires and clinical visits.

Ethical approval for the ALSPAC study was obtained from the ALSPAC Law and Ethics Committee (ALEC; IRB00003312) and Local Research Ethics Committees. Informed consent for the use of data collected via questionnaires and clinics was obtained from participants following the recommendations of the ALSPAC Ethics and Law Committee at the time. At age 18, study children were sent ‘fair processing’ materials describing ALSPAC's intended use of their health and administrative records and were given clear means to consent or object via a written form. Data were not extracted for participants who objected, or who were not sent fair processing materials. In order to conduct this research study, members of our research team (JP, RJL) obtained funding from The 10.13039/501100000862Sir Halley Stewart Trust (Grant reference: 809) to make a specific request to ALSPAC for the insertion of the European Health Literacy Survey Questionnaire-short version (HLS-EU-Q16) within the ALSPAC Life@25+ Questionnaire and further requested access to use relevant ALSPAC data for analysis via submission of a research proposal to the ALSPAC Executive Board and linkage to the ALSPAC Data Access Team with approval granted by ALEC (Ref 56,121, October 2017).

This research focused on numerous factors that may be predictive of later adult low health literacy. It was informed by Forrest et al.‘s 4D model (Forrest et al., 1997; Guo et al., 2018) that considers development change (changes in cognitive ability), dependency (parental and caregiver influence), differential (children/adolescents experience different illnesses), and demographic (household and environmental deprivation), and Sørensen et al.'s (2012) conceptual model of adult health literacy that includes societal and environmental (e.g. demographic), personal (e.g. education) and situational (e.g. family effects) influences. In support to these choices Marmot has highlighted the influence of maternal and childhood factors in determining health (Marmot et al., 2020). Where these data were available in the dataset we included them in the analysis i.e. housing (owner-occupier status), education level, disability (maternal health) and environment (Index of Multiple Deprivation). These factors (as described in Table 1) were identified within the following ALSPAC cohort stages approximating to children aged 5–8 years, 9–11.5 years, and the outcome measure of health literacy at participant aged 25 years. In total 9989 eligible individuals were invited to complete the ALSPAC Life@25+ Questionnaire. The population for this study was all adults who responded to the ALSPAC Life@25+ questionnaire and completed the health literacy questions (n = 4248) (see Figure A1 in the online supplementary material).

**Table 1:** Potential predictive factors for adult low health literacy.

Domain	Measures	Child age when assessed	Supportive literature
Child educational ability	Entry assessment score (Language, reading, writing and mathematics)	5–7 years	Alati et al., 2013; Meadows et al., 2008; Sabates et al., 2011
	Key Stage 1 score (Reading, writing and mathematics)	5–7 years	
	Key Stage 2 English	7–11 years	
	Key Stage 2 Maths	7–11 years	
	Key Stage 2 Science	7–11 years	

Child speech and language ability	Assessment of phrase length, sentence structure, use of grammar	9.5 years	Clegg et al., 2015; Schoemaker et al., 2013; Skuse et al., 2009

Child general health	Assessment of parental report of child's general health in past year	6.5 and 11 years	Paget et al. (2018)

Child healthcare engagement	Count of number of different conditions child saw doctor for in past year	6.5 years	Beale et al., 2010; Hay et al., 2005

Child sleep problems	Questions assessing difficulty going to sleep, presence of nightmares, incomplete sleep (getting up after few hours)	6.5 and 9 years	Blair et al., 2012; Humphreys et al., 2014; Lereya et al., 2017; Scott et al., 2013

Child mental health	Strengths and Difficulties Questionnaire (SDQ): Externalising	6.5 and 11.5 years	Achenbach et al., 2008; Andreucci et al., 2021; Goodman, 2001; Green et al., 2005; Mellor, 2005; Moriwaki & Kamio, 2014
	Strengths and Difficulties Questionnaire (SDQ): Internalising	6.5 and 11.5 years	
	Short Mood and Feelings Questionnaire: Depression	9 and 11.5 years	Angold et al., 1995; Culpin et al., 2015; Easey et al., 2020; Sharp et al., 2006

Environmental and Maternal factors	Index of Multiple Deprivation 2000	8 years	Noble et al., 2006; Yakubovich et al., 2020
	Mothers' educational level	5 years	Burstyn et al., 2010; Haynes et al., 2008; Plant et al., 2017
	Mothers' home ownership	5 years	
	Mothers' physical health	5 years	
	Mothers' anxiety and nerves	6 years	
	Mothers' depression	6 years	

Study data for the ALSPAC Life@25+ questionnaire were collected and managed using REDCap electronic data capture tools hosted at the University of Bristol (Harris et al., 2009). REDCap (Research Electronic Data Capture) is a secure, web-based software platform designed to support data capture for research studies.

### 2.2 Adult health literacy

The HLS-EU-Q16 is a short form of the HLS-EU-Q47 questionnaire (Sørensen et al., 2013) and has demonstrated good approximation of the full 47-item version, with a high correlation (r = 0.82) between the HLS-EU-Q16 and the general health literacy score of the HLS-EUQ47, and a 75.8% concurrent classification of respondents as having insufficient, limited, and sufficient health literacy (Pelikan et al., 2014; Vandenbosch et al., 2016). The HLS-EU-Q16 which can be found in the online supplementary material asks how easy it is for the participant to find, understand and use information related to health, illness, and medical care. Items are rated on a four-point Likert scale ranging from ‘very easy’ to ‘very difficult’. Responses of ‘very easy’ or ‘easy’ were coded as 1, and ‘difficult’ or ‘very difficult’ coded as 0; if one or two responses (out of 16) to the questionnaire were missing, the scores were calculated based on the observed responses to the other items, this assumed that an imperfect score differing from the true score by one or two units was a good proxy of the true score (Gustafsdottir et al., 2020).

### 2.3 Childhood factors

ALSPAC has collected data annually from birth to age 25 and measured various factors in childhood that may be associated low health literacy in adults; this study focused particularly on modifiable factors as these can form potential intervention targets in future studies. Table 1 outlines the potential predictive factors used within this study. Briefly, these factors fall within seven broader domains that cover: child's educational ability as covered by compulsory national curriculum tests in the UK (Foundation entry assessment and Key Stage Standard Assessment Tests), child's speech and language ability, child's general health, child's healthcare engagement, child sleep problems, child's mental health, environmental factors including neighbourhood deprivation, and maternal factors such as mother's educational attainment, mother's home ownership status, and mother's physical and mental health. Supportive literature is also included in Table 1 to document previous usage of the measures with ALSPAC and/or previous validation studies of those measures. Please refer to Table A1 in the online supplementary material for a more detailed description of the variables and usage within the analysis in this study.

### 2.4 Statistical analysis

Responses to the HLS-EU-Q16 were summed to give an overall health literacy score of 0–16. For interpretation, this score was categorised as insufficient (0–8), limited (9–12), and sufficient (13–16) health literacy following previous methodology (Vandenbosch et al., 2016) to define the primary outcome. Predictive factors in childhood were described by level of adult health literacy using mean and standard deviation for continuous variables and frequency and percentage for categorical variables. Univariate proportional odds logistic regression models were fitted to examine the relationship between each childhood factor described in Table 1 and Table A1 in the online supplementary material and the level of health literacy at age 25 years. The proportional odds assumption was checked for each model by comparing the empirical cumulative odds of health literacy; when this assumption was not tenable multinomial logistic regression models were fitted. Odds ratios and 95% confidence intervals (CI) comparing the odds of a higher health literacy category (limited or sufficient *vs* low) or the odds ratios (95% CI) comparing the odds of a category (limited *vs* low and sufficient *vs* low) were reported for the proportional odds and multinomial logistic regression models respectively. Goodness of fit was assessed through the deviance statistic. All models were fitted using maximum likelihood estimation based on all available data from those participants who completed the HLS-EU-Q16. This assumes a missing at random mechanism, patterns of missingness were explored. Participant characteristics and percentage of missingness across measurements were compared with those from the full ALSPAC cohort and the sample of participants who were invited to complete the ALSPAC Life@25+ Questionnaire. Analyses were performed using the statistical software R (R Core Team, 2021).

## 3 Results

In total ALSPAC Life@25+ Questionnaire was sent to 9989 individuals. It was returned by 4393 individuals, 123 (3%) of whom did not fill in section D of the questionnaire which contains the HLS-EU-Q16, 24 questionnaires (0.5%) had missing responses to 1 (16/4393) or 2 items (8/4393) for which health literacy scores were calculated using the observed remaining 14 or 15 items and 22 (0.5%) had 3 or more missing responses which were excluded from the analysis, leaving data from 4248 individuals for analysis.

### 3.1 Descriptive analysis

Table 2 describes the participant's characteristics overall and by levels of adult health literacy. More than three quarters (77%; N = 3277) of those who completed the HLS-US-Q16 had sufficient health literacy, 18% (744) had limited and 5% (227) had insufficient health literacy. The cohort included 66.2% female participants. Three quarters of the mothers had further or higher education level when the child was 5 years of age. A small percentage (2.5%) had no educational qualification. Most mothers (87%) owned their home with a lower percentage (79%) seen in those whose child had insufficient literacy in adulthood. The household 2000 IMD score quintile, measured when the child was 8 years of age, was 1 (most deprived area) for 35% of the sample and 4 or above (least deprived area) for 27% of the participants, slightly higher levels of deprivation were seen for those where the participant had insufficient health literacy (30.4% vs 35.4% and 34.9% in limited and sufficient health literacy respectively).

**Table 2:** Participant characteristics by level of health literacy.

HLS-EU-Q16 ordinal score	Insufficient, 227 (5%)	Limited, 744 (18%)	Sufficient, 3277 (77%)	Total, 4248 (100%)
Sex, Female, N (%)	151 (66.5)	499 (67.1)	2162 (66.0)	2812 (66.2)
Entry assessment score (Language, reading, writing and mathematics), Mean (SD)	12.7 (3.5)	14.0 (3.0)	13.9 (3.0)	13.9 (3.1)
Key Stage 1 score (Reading, writing and mathematics), Mean (SD)	9.5 (4.0)	10.8 (3.2)	10.8 (3.2)	10.7 (3.3)
Key Stage 2 score, Mean (SD)				
English	4.1 (1.1)	4.4 (0.7)	4.4 (0.7)	4.4 (0.7)
Mathematics	4.2 (1.1)	4.4 (0.7)	4.3 (0.7)	4.3 (0.8)
Science	4.4 (0.9)	4.6 (0.6)	4.6 (0.6)	4.6 (0.6)
Child's speech is mostly just two or three word phrases (9y 7m), True, N (%)	<5 (<5)	<5 (<5)	<5 (<5)	8 (0.2)
Child can produce long and complicated sentences (9y 7m), True, N (%)	164 (95.3)	576 (98.1)	2625 (98.9)	3365 (98.6)
Child tends to leave out words and grammatical endings (9y 7m), True, N (%)	8 (4.7)	7 (1.2)	25 (0.9)	40 (1.2)
Child sometimes makes mistakes with pronouns (9y 7m), True, N (%)	25 (14.5)	39 (6.7)	189 (7.2)	253 (7.4)
Assessment of child's health in past year, Very healthy or healthy, N (%)				
6 y 9 m	159 (96.4)	555 (98.4)	2353 (98.4)	3067 (98.3)
11 y 8 m	158 (98.8)	549 (99.1)	2397 (98.0)	3104 (98.2)
No. Different conditions child saw doctor for in past year (6y 9m), Mean (SD)	1.3 (1.9)	1.1 (1.6)	1.1 (1.7)	1.1 (1.7)
Child had difficulty going to sleep in past year, Yes, N (%)				
6 y 9 m	112 (65.9)	392 (66.2)	1635 (64.3)	2139 (64.7)
9 y 7 m	99 (57.2)	314 (54.0)	1377 (52.1)	1790 (52.7)
Child had nightmares in past year, Yes, N (%)				
6 y 9 m	93 (55.7)	309 (52.4)	1251 (49.4)	1653 (50.3)
9 y 7 m	46 (27.1)	165 (29.0)	724 (27.8)	935 (28.0)
Child got up after few hrs sleep in past year, Yes, N (%)				
6 y 9 m	24 (14.1)	55 (9.4)	274 (10.8)	353 (10.7)
9 y 7 m	14 (8.1)	28 (4.8)	142 (5.4)	184 (5.4)
SDQ externalising construct score, Mean (SD)				
6 y 9 m	5.1 (3.5)	4.7 (3.0)	4.4 (3.1)	4.5 (3.1)
11 y 8 m	4.2 (3.4)	3.4 (2.7)	3.4 (2.9)	3.5 (2.9)
SDQ externalising construct, score above 90th percentile, N (%)				
6 y 9 m	24 (14.0)	40 (6.8)	172 (6.8)	236 (7.2)
11 y 8 m	22 (13.4)	48 (8.3)	221 (8.7)	291 (8.8)
SDQ internalising construct score, Mean (SD)				
6 y 9 m	3.3 (2.9)	2.7 (2.7)	2.4 (2.4)	2.5 (2.5)
11 y 8 m	3.4 (3.4)	2.8 (2.8)	2.4 (2.6)	2.5 (2.7)
SDQ internalising construct, score above 90th percentile, N (%)				
6 y 9 m	32 (18.7)	83 (14.0)	290 (11.4)	405 (12.2)
11 y 8 m	37 (22.7)	86 (14.9)	289 (11.3)	412 (12.5)
SMFQ depression score, Mean (SD)				
9 y 7 m	2.8 (3.3)	2.7 (2.9)	2.3 (3.0)	2.4 (3.0)
11 y 8 m	2.9 (3.8)	2.4 (3.2)	2.1 (3.0)	2.2 (3.1)
SMFQ depression, score above 90th percentile, N (%)				
9 y 7 m	21 (12.4)	71 (12.2)	223 (8.4)	315 (9.3)
11 y 8 m	24 (14.6)	72 (12.5)	255 (10.0)	351 (10.7)
2000 IMD score quintiles				
1	45 (30.4)	186 (35.4)	827 (34.9)	1058 (34.8)
2	24 (16.2)	107 (20.4)	477 (20.1)	608 (20.0)
3	28 (18.9)	91 (17.3)	438 (18.5)	557 (18.3)
4	30 (20.3)	88 (16.8)	347 (14.6)	465 (15.3)
5	21 (14.2)	53 (10.1)	281 (11.9)	355 (11.7)
Mother's educational level				
No educational qualification	6 (3.4)	13 (2.2)	64 (2.5)	83 (2.5)
O-levels, CSE/GCSE	39 (22.4)	113 (19.0)	516 (20.2)	668 (20.1)
Further education	75 (43.1)	283 (47.6)	1142 (44.6)	1500 (45.1)
Higher education	54 (31.0)	186 (31.3)	837 (32.7)	1077 (32.4)
Mother's home ownership status, Owned, N (%)	143 (79.0)	534 (88.6)	2240 (86.7)	2917 (86.6)
Mother's description of current health, Well and healthy, N (%)	163 (91.6)	553 (93.4)	2412 (94.1)	3128 (93.8)
Mother has had anxiety or ‘nerves’ in last 3 years, Yes, N (%)	55 (30.7)	119 (20.2)	524 (20.5)	698 (21.0)
Mother has had depression in last 3 years, Yes, N (%)	53 (29.6)	130 (22.0)	518 (20.3)	701 (21.1)

HLS-EU-Q16: Health Literacy Survey Questionnaire-short version; SD: Standard deviation; SDQ: Strengths and Difficulties Questionnaire; SMFQ: Short Mood and Feelings Questionnaire; IMD: Index of Multiple Deprivation; O-level: The lowest of three levels of standardised British examinations in a secondary school subject; CSE: A qualification in a specific subject formerly taken by school students aged 14–16; it was the lowest of three levels of standardised British examinations in a secondary school subject at a level below O-level. It was replaced in 1988 by the GCSE; GCSE: A qualification in a specific subject typically taken by school students aged 14–16.

### 3.2 Regression models for adult health literacy score

**Fig. 1:**
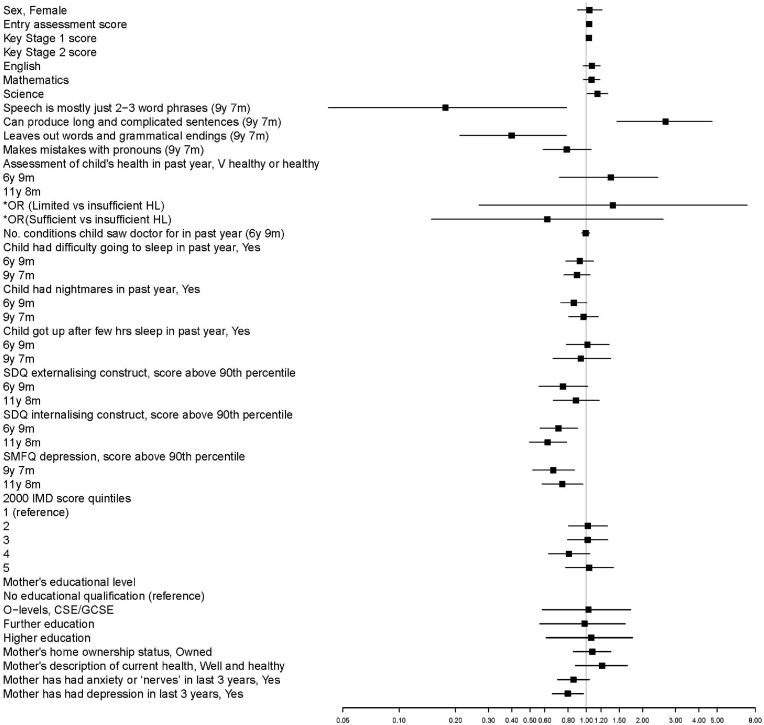
Odds ratios (95% confidence intervals) of higher levels of health literacy × OR: Odds ratios estimated using multinomial logistic regression; SDQ: Strengths and Difficulties Questionnaire; SMFQ: Short Mood and Feelings Questionnaire; IMD: Index of Multiple Deprivation; CSE: A qualification in a specific subject formerly taken by school students aged 14–16; it was the lowest of three levels of standardised British examinations in a secondary school subject, at a level below O-level. CSE and O-level were replaced in 1988 by the GCSE; GCSE is a qualification in a specific subject typically taken by school students aged 14–16.

Table 3 and Fig. 1 show the estimated odds ratios of higher levels of adult health literacy and 95% CIs for variables across the domains described in Table 1. There was no evidence of differential health literacy levels between male and female participants. For the child's educational ability (entry assessment and key stage scores) there was a small percentage increase in odds of sufficient adult health literacy for those who scored higher on entry assessment score (age 5–7 years) (+4%) and key stage 1 score (+3%). There was no evidence of an association based on Key Stage 2 (age 7–11 years) English or Maths, however an association (+15%) was shown for sufficient adult health literacy based on better performance in science. Considering child speech and language ability (child age 9 years and 7 months), results show evidence of a decrease in odds of sufficient adult health literacy if speech is limited to 2–3 word phrases (−82%), a reduction (−60%) of sufficient adult health literacy for children who leave out words or grammatical endings, with evidence of a weaker trend in a reduction of odds (−21%) for children who make mistakes with pronouns. Conversely there was a large increase (+2.7-fold) of sufficient adult health literacy for children who can produce long and complicated sentences.

**Table 3:** Estimated odds ratios (95% confidence intervals) of higher levels of health literacy.

Explanatory variable	Odds ratio	95% Confidence interval
Sex, Female	1.04	0.9 to 1.21
Entry assessment score (Language, reading, writing and mathematics)	1.04	1.01 to 1.07
Key Stage 1 score (Reading, writing and mathematics)	1.03	1.01 to 1.06
Key Stage 2 score		
English	1.07	0.97 to 1.19
Mathematics	1.07	0.97 to 1.18
Science	1.15	1.01 to 1.3
Speech is mostly just 2–3 word phrases (9y 7m)	0.18	0.04 to 0.78
Can produce long and complicated sentences (9y 7m)	2.66	1.46 to 4.71
Leaves out words and grammatical endings (9y 7m)	0.40	0.21 to 0.78
Makes mistakes with pronouns (9y 7m)	0.79	0.59 to 1.06
Assessment of child's health in past year, V healthy or healthy		
6 y 9 m	1.35	0.72 to 2.41
11 y 8 m		
aOR (Limited vs insufficient HL)	1.39	0.27 to 7.23
aOR (Sufficient vs insufficient HL)	0.62	0.15 to 2.57
No. Conditions child saw doctor for in past year (6y 9m)	0.99	0.95 to 1.04
Child had difficulty going to sleep in past year, Yes		
6 y 9 m	0.92	0.78 to 1.09
9 y 7 m	0.89	0.76 to 1.05
Child had nightmares in past year, Yes		
6 y 9 m	0.86	0.73 to 1.01
9 y 7 m	0.97	0.81 to 1.16
Child got up after few hrs sleep in past year, Yes		
6 y 9 m	1.01	0.78 to 1.33
9 y 7 m	0.94	0.66 to 1.35
SDQ externalising construct, score above 90th percentile		
6 y 9 m	0.75	0.56 to 1.02
11 y 8 m	0.88	0.67 to 1.17
SDQ internalising construct, score above 90th percentile		
6 y 9 m	0.71	0.57 to 0.9
11 y 8 m	0.62	0.5 to 0.78
SMFQ depression, score above 90th percentile		
9 y 7 m	0.67	0.52 to 0.86
11 y 8 m	0.74	0.58 to 0.96
2000 IMD score quintiles		
1	Reference	
2	1.02	0.8 to 1.3
3	1.02	0.79 to 1.31
4	0.81	0.63 to 1.04
5	1.04	0.77 to 1.4
Mother's educational level		
No educational qualification	Reference	
O-levels, CSE/GCSE	1.03	0.58 to 1.73
Further education	0.98	0.56 to 1.62
Higher education	1.06	0.61 to 1.77
Mother's home ownership status, Owned	1.08	0.85 to 1.36
Mother's description of current health, Well and healthy	1.21	0.88 to 1.66
Mother has had anxiety or ‘nerves’ in last 3 years, Yes	0.85	0.7 to 1.04
Mother has had depression in last 3 years, Yes	0.80	0.66 to 0.96

a Estimated OR using a multinomial distribution; SD: Standard deviation; SDQ: Strengths and Difficulties Questionnaire; SMFQ: Short Mood and Feelings Questionnaire; IMD: Index of Multiple Deprivation; O-level: The lowest of three levels of standardised British examinations in a secondary school subject; CSE: A qualification in a specific subject formerly taken by school students aged 14–16; it was the lowest of three levels of standardised British examinations in a secondary school subject, at a level below O-level. It was replaced in 1988 by the GCSE; GCSE: A qualification in a specific subject typically taken by school students aged 14–16.

For child general health (assessed over the past year at age 6 year 9 months, and 11 years and 8 months) there was no evidence of association, similarly there was no evidence of association found between the number of health conditions the child saw a doctor for over the past year at age 6 years and 9 months and later adult health literacy. Analysis of the presence of child sleep problems including difficulty going to sleep, presence of nightmares, and getting up after a few hours of sleep (at ages 6 years and 9 months, and 9 years and 7 months) showed no association with adult health literacy.

Consideration of child mental health shows weak evidence of a trend for a reduction in odds of sufficient adult health literacy for children with high externality at age 6 years and 9 months (−25%) and 11 years and 8 months (−12%), with evidence of a reduction in odds for children who score high on internalising at age 6 years and 9 months (−29%) and at age 11 years and 8 months (−38%). Similarly, there was a consistent reduction in odds of sufficient adult health literacy for children who report high levels of depression at age 9 years and 7 months (−33%) and at 11 years and 8 months (−26%).

Exploration of potential wider determinants at childhood on adult health literacy shows no association dependent on whether the child's mother was a homeowner or not, no association with adult health literacy dependent on the mothers' level of education, and no association dependent on the child's index of deprivation. Examination of mothers' health status showed weak evidence of a trend of increase (+21%) in sufficient adult health literacy if mother indicated being well and healthy. There was also weak evidence of a trend effect of decreasing odds (−15%) of sufficient adult health literacy if the mother indicated presence of anxiety, with a decrease in odds (−20%) if the mother indicated presence of depression.

The results of the assessment of potential selection bias from completers which was undertaken by comparing the participant characteristics and missingness in this study cohort with those of the cohort of participants who were invited to complete the ALSPAC@25+ Questionnaire show responders had slightly more females, had higher overall educational ability, reported less psychological problems (e.g. internalising, externalising, depression), had similar levels of health and sleep problems. Responders also had slightly higher levels of deprivation, with mothers reporting better educational outcomes and home ownership but with similar levels of maternal health (for full details see online supplementary material, Table A2). The assessment of generalisability of our study cohort through the comparison of the participant characteristics and the full ALSPAC cohort can be found in the online supplementary material.

## 4 Discussion

This study, analysing data from a large prospective UK birth cohort (assessed at multiple timepoints measuring a wide range of potential predictive factors), has produced new evidence showing that not only child's educational ability, but also child speech and language ability, child mental health status, and the presence of maternal depression are associated with later adult health literacy. This study contributes to our growing knowledge of the development of health literacy and indicates factors within childhood that may provide markers for future intervention development.

### 4.1 Comparison with existing literature

The results of this study are supportive of both Forrest et al.'s model (1997) of child health literacy and Sørensen et al.'s (2012) conceptual model of adult health literacy from the perspective of general cognitive and educational ability, with evidence of important effects found in this study for child educational ability and later adult health literacy. In addition, these educational ability findings are reflective of other longitudinal cohort studies such as Clouston et al.'s (2017) finding that high school graduate ranking was predictive of later low adult health literacy in a US study, and Richards et al.'s (2009) use of birth cohort data from the UK which linked child educational ability at age 8 years to later general literacy. However, this current study has given greater insight by demonstrating only small effects of educational ability at a younger age (age 5 to 7), but this effect is stronger (particularly in the topic of science) at older ages. Furthermore, the strongest and most consistent effect is the child's speech and language capability at age 9.5 years. These exploratory findings may indicate where health literacy interventions in schools should focus for maximum effect, but further study should be undertaken.

Another feature within health literacy models (Forrest et al., 1997; Sørensen et al., 2012; van der Heide et al., 2016), and the cohort studies mentioned above (Clouston et al., 2017; Richards et al., 2009), is the consistent effect of the immediate environment and family influence, predominantly the influence of parental socio-economic status and markers of deprivation. This current study did not find strong associations across these components, even though the study included three markers *i.e.* IMD score, mother's educational attainment, and mother's home ownership status. Examination of potential trend effects show a small increase in odds for sufficient adult health literacy if the mother is a homeowner and if the mother has higher educational attainment, but there is no effect shown for neighbourhood level deprivation (IMD). There may be several reasons: Firstly, when compared with a data sample from the National Pupil Database, children in ALSPAC have a higher socio economic status at age 16, are more likely to be White, and less likely to be eligible for free school meals (Boyd et al., 2013) and so the effects reported here may be underestimates given that those from non-White backgrounds and those who have lower social economic status are associated with low health literacy (Rowlands et al., 2015). Secondly the use of IMD data gives a neighbourhood approximation of deprivation, and it may be the actual household markers would have given greater accuracy. Thirdly, this study only included mothers' educational attainment and therefore did not include father, partner, or additional family information which may have given greater accuracy.

A key finding within this current study is the consistent effect of child low mood (internalising), child depression, and maternal depression, in relation to later adult insufficient and limited health literacy. Robust evidence exists that shows the consequences of mood in child and parents on later adult health and wellbeing (Bellis et al., 2019; Jones et al., 2018), however examination of Sørensen et al.'s (2012) comprehensive integration of 12 conceptual models of health literacy show that none consider mood as an antecedent to health literacy status, similarly a framework for understanding health literacy within adolescents (Manganello, 2008) also does not consider mood. Our findings suggest this is an important omission within the current literature and more research is required to understand the effects of mood (in child and mother) on the development of health literacy.

### 4.2 Strengths and limitations

A strength of this study is the use of data from a large well established prospective birth cohort with long term follow-up that enabled temporal examination of factors within childhood associated with later adult health literacy. An additional strength is the inclusion of a validated measure of adult health literacy within the ALSPAC Life@25+ questionnaire (as requested by members of our research team, JP, RJL) for the purpose of this study. A further strength is the availability and use of various measures within childhood relevant to current theoretical models of health literacy development, and that these measures are at different stages of childhood to facilitate greater understanding of development. Limitations include that ALSPAC is not typical of the general population with a lower percentage of minority ethnic groups and those from low economic status (which are likely to have lower health literacy, Rowlands et al., 2015) which may have led to underestimations of effects. Consideration of these potential effects arising from differences between the ALSPAC cohort and the general population show that results from the HLS-US-Q16 measure indicated 23% of the cohort had insufficient or limited health literacy compared with a review of 85 studies estimating a prevalence of 26% (Paasche-Orlow et al., 2005), therefore any differences are likely to have minimal effect on our reported findings. A further limitation with this study is the lack of information of parental influences/attitudes on health and parental health literacy, this is a consistent feature of various conceptual models (Sørensen et al., 2012) and further information is needed on the interaction effects of parents and children (e.g. paternal and maternal influences, birth order, family size, non-parental carers and step parents) with regard the development of later adult health literacy. Additionally, whilst this study used a validated measure of adult health literacy, it was restricted to self-report and the inclusion of face-to-face interviews and/or the use of vignettes may have given richer detail to the findings. Finally, there is potential selection bias arising from the analysis of data from participants who had higher rates of completion of questionnaires.

### 4.3 Interpretation and implications

This study's results have identified several factors within childhood that are associated with later adult health literacy status. Having the potential to identify those at higher risk of later low health literacy could be of future health, social, and economic benefit. Our results support previous literature in the identification of child educational ability as an important factor but have now expanded on this general finding by adding information on the specific components of educational ability that appear linked to later adult health literacy and highlight that this may be best manifest by the child's speech and language capability at age 9 years as one area with particularly strong effects. These educationally based findings may indicate a useful marker to identify children at potential risk, and future research could explore potential interventions within school settings to help build adequate health literacy within identified at risk groups. In addition, this study has identified child and maternal mental health as factors associated with later development of limited health literacy and future research should consider what potential mechanisms might explain this link. Additionally, as this current study was explorative and observational, with the purpose to identify potential factors in childhood associated with adult health literacy, future studies should look to develop models that can consider interactions between variables to disentangle their relative contribution as well as consider potential mediator and moderator effects. Alternative study approaches such as family or twin-designs could be useful to address observed and unobserved confounding and would be avenues for further research.

## Funding statement

The UK Medical Research Council and Wellcome (Grant ref: 217065/Z/19/Z) and the University of Bristol provide core support for ALSPAC. This publication is the work of the authors and I. Solis-Trapala and J. Protheroe will serve as guarantors for the contents of this paper. A comprehensive list of grants funding is available on the ALSPAC website (http://www.bristol.ac.uk/alspac/external/documents/grant-acknowledgements.pdf); This research was specifically funded by The Sir Halley Stewart Trust (Grant reference: 809). G. Rowlands’ time is funded by the National Institute for Health Research (NIHR) Applied Research Collaboration (ARC) North East and North Cumbria (NIHR200173). The views expressed are those of the authors and not necessarily those of the NIHR or the Department of Health and Social Care. The funding sources had no role in the analysis and interpretation of data, in the writing of the report or in the decision to submit the article for publication.

Health Literacy UK group (not for profit organisation) funds the publication of this research paper.

## Ethical statement

This study utilises data gathered from the Avon Longitudinal Study of Parents and Children (ALSPAC) study (Boyd et al., 2013; Fraser et al., 2013). ALSPAC is a longitudinal birth cohort which enrolled 14,541 pregnant women who resided in one of three Bristol-based health districts in England with expected delivery date between April 1, 1991 and December 31, 1992.

Of these initial pregnancies, there were 14,062 live births and 13,988 children were alive at 1 year of age. When the oldest children were approximately 7 years old, the initial sample was bolstered with eligible cases who had failed to join the study originally. Therefore, there is data available for more than the 14,541 pregnancies mentioned above (Northstone et al., 2019). These children, their mothers and partners have been followed up regularly over the past three decades using a combination of postal questionnaires and clinical visits (“About | Avon Longitudinal Study of Parents and Children | University of Bristol”). Please note that the study website contains details of all the data that is available through a fully searchable data dictionary and variable search tool (“Explore data and samples | Avon Longitudinal Study of Parents and Children | University of Bristol”).

Ethical approval for the ALSPAC study was obtained from the ALSPAC Law and Ethics Committee (ALEC; IRB00003312) and Local Research Ethics Committees. Informed consent for the use of data collected via questionnaires and clinics was obtained from participants following the recommendations of the ALSPAC Ethics and Law Committee at the time. At age 18, study children were sent ‘fair processing’ materials describing ALSPAC's intended use of their health and administrative records and were given clear means to consent or object via a written form. Data were not extracted for participants who objected, or who were not sent fair processing materials.

## CRediT authorship contribution statement

**I. Solis-Trapala:** Data curation, Formal analysis, Investigation, Methodology, Writing – original draft, Writing – review & editing. **P. Campbell:** Conceptualization, Investigation, Methodology, Writing – original draft, Writing – review & editing. **K.M. Dunn:** Investigation, Methodology, Writing – review & editing. **R.J. Lacey:** Conceptualization, Data curation, Funding acquisition, Investigation, Methodology, Writing – original draft, Writing – review & editing. **G. Rowlands:** Conceptualization, Investigation, Methodology, Writing – review & editing. **J. Protheroe:** Conceptualization, Funding acquisition, Investigation, Methodology, Writing – original draft, Writing – review & editing.

## Declaration of competing interest

None.

## Data Availability

This study utilised data collected from the ALSPAC study. The study website contains details of all the data that is available through a fully searchable data dictionary and variable search tool.
