# Supplementary material for: Are childhood factors predictive of adult health literacy? A longitudinal birth cohort analysis
Source: SSM Popul Health. 2023 May 8;23:101426. doi: 10.1016/j.ssmph.2023.101426 (PMC10220279; doi:10.1016/j.ssmph.2023.101426)
Supplement: Multimedia component 1 [file mmc1.docx]

**Online supplementary material**

Contents

[Flowchart of the ALSPAC study cohort and timeline of factors linked to health literacy 2](#_Toc115862270)

[European Health Literacy Survey Questionnaire- short version 3](#_Toc115862271)

[Description of variables used in the study 4](#_Toc115862272)

[Comparison of participant characteristics and missingness patterns of this study cohort with the cohort of participants who were invited to complete the ALSPAC Life@25+ Questionnaire and the full ALSPAC sample 6](#_Toc115862273)

# Flowchart of the ALSPAC study cohort and timeline of factors linked to health literacy

Figure A1 describes the study data flow comprising the cohort of participants who completed the European Health Literacy Survey Questionnaire- short version (HLS-EU-Q16) at 25 years of age, and longitudinal measurements of childhood factors potentially linked to adult health literacy.

**Figure A1. Flowchart of the ALSPAC cohort and timeline of measurement of the domains**


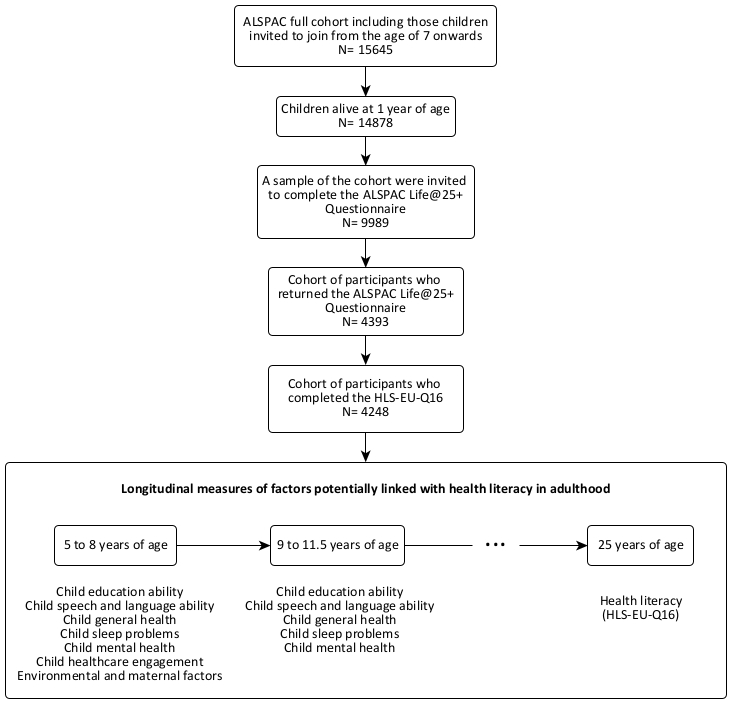


# European Health Literacy Survey Questionnaire- short version

The HLS-EU-Q16 formed part of the ALSPAC Life@25+ Questionnaire and is shown below.

**How easy or difficult is it for you to:**

|  | Very easy | Easy | Difficult | Very difficult |
| --- | --- | --- | --- | --- |
| a. Find information on treatments of illnesses that concern you? |  |  |  |  |
| b. Find out where to get professional help when you are ill? |  |  |  |  |
| c. Understand what your doctor says to you? |  |  |  |  |
| d. Understand your doctor's or pharmacist's instruction on how to take a prescribed medicine? |  |  |  |  |
| e. Judge when you may need to get a second opinion from another doctor? |  |  |  |  |
| f. Use information the doctor gives you to make decisions about your illness? |  |  |  |  |
| g. Follow instructions from your doctor or pharmacist? |  |  |  |  |
| h. Find information on how to manage mental health problems like stress or depression? |  |  |  |  |
| i. Understand health warnings about behaviour such as smoking, low physical activity and drinking too much? |  |  |  |  |
| j. Understand why you need health screenings? |  |  |  |  |
| k. Judge if the information on health risks in the media is reliable? |  |  |  |  |
| l. Decide how you can protect yourself from illness based on information in the media? |  |  |  |  |
| m. Find out about activities that are good for your mental well-being? |  |  |  |  |
| n. Understand advice on health from family members or friends? |  |  |  |  |
| o. Understand information in the media on how to get healthier? |  |  |  |  |
| p. Judge which everyday behaviour is related to your health? |  |  |  |  |

#

# Description of variables used in the study

**Table A1. Description of variables used in the statistical analysis**

| **Domain** | **Measures** | **Participant age when assessed** | **Description of variable used in the statistical analysis** |
| --- | --- | --- | --- |
| Health literacy | HLS-EU-Q16 score | 25 years | Ordinal variable derived from total score (range 0 to 16) as:  Insufficient: Total score 0 to 8  Limited: Total score 9 to 12  Sufficient: Total score 13 to 16 |
|  |  |  |  |
| Demographic | Sex of child |  | Categorical variable: “Female”, “Male” |
|  |  |  |  |
| Child educational ability | Entry assessment score (Language, reading, writing and mathematics) | 5 to 7 years | Prorated* score derived from the sum of scores obtained in language, reading, writing and mathematics tests, range 0 to 20 |
|  | Key Stage 1 score (Reading, writing and mathematics) |  | Prorated* score derived from the sum of the scores obtained in reading, writing and mathematics tests, range 0 to 15 |
|  | Key Stage 2 English | 7 to 11 years | Ordinal scores: -1= working below the level assessed by the test, or disapplied, 0=no test level awarded, or absent, 1= achieved level 1, 2=achieved level 2, 3= achieved level 3, 4=achieved level 4, 5=achieved level 5, 6=achieved level 6 |
|  | Key Stage 2 Maths |  |  |
|  | Key Stage 2 Science |  |  |
|  | | |  |
| Child speech and language ability | Child's speech is mostly just two- or three-word phrases | 9.5 years | Categorical variables: “True” or “not true” |
|  | Child can produce long and complicated sentences |  |  |
|  | Child tends to leave out words and grammatical endings |  |  |
|  | Child sometimes makes mistakes with pronouns |  |  |
|  | | |  |
| Child general health | Assessment of parental report of child’s general health in past year | 6.5 and 11 years | Categorical variables: “Very healthy or healthy”, “Sometimes or almost always unwell” |
|  | | |  |
| Child healthcare engagement | Count of number of different conditions child saw doctor for in past year | 6.5 years | Count variable |
|  | | |  |
| Child sleep problems | Child had difficulty going to sleep in past year | 6.5 and 9.5 years | Categorical variables: “Yes”, “No” |
|  | Child had nightmares in past year |  |  |
|  | Child got up after few hours’ sleep in past year |  |  |
|  | | |  |
| Child mental health | Strengths and Difficulties Questionnaire (SDQ) Externalising | 6.5 and 11.5 years | Prorated* score, range 0 to 20. This score was dichotomised using the 90% percentile of the sample as threshold because this corresponds to an accepted clinical level cut point** |
|  | Strengths and Difficulties Questionnaire (SDQ) Internalising |  | Prorated* score, range 0 to 20. This score was dichotomised using the 90% percentile of the sample as threshold because this corresponds to an accepted clinical level cut point** |
|  | Short Mood and Feelings Questionnaire Depression | 9 and 11.5 years | Prorated* score, range 0 to 26. This score was dichotomised using the 90% percentile of the sample as threshold because this corresponds to an accepted clinical level cut point** |
|  | | |  |
| Environmental and Maternal factors | Index of Multiple Deprivation (IMD) 2000 | 8 years | Categorical variable for quintiles, IMD is a measure of relative deprivation for small, fixed geographic areas of the UK. IMD classifies these areas into five quintiles based on relative disadvantage, with quintile 1 being the most deprived and quintile 5 being the least deprived |
|  | Mothers’ educational level | 5 years | Categorical variable: “No educational qualification”, “O-levels”, “CSE/GCSE”,  “Further education”, “Higher education”.  CSE: A qualification in a specific subject formerly taken by school students aged 14–16; it was the lowest of three levels of standardised British examinations in a secondary school subject, at a level below O-level. CSE and O-level were replaced in 1988 by the GCSE; GCSE is a qualification in a specific subject typically taken by school students aged 14–16 |
|  | Mothers’ home ownership | 5 years | Categorical variable: “Owned”, “Not owned” |
|  | Mothers’ physical health | 5 years | Categorical variable: “Well and healthy”, “often unwell” |
|  | Mothers’ anxiety and nerves in last 3 years | 6 years | Categorical variable: “Yes”, “No” |
|  | Mothers’ depression in last 3 years | 6 years | Categorical variable: “Yes”, “No” |

*Prorated scores were scaled to account for missing item responses; HLS-EU-Q16: European Health Literacy Survey Questionnaire- short version. ** Mellor D. Normative data for the Strengths and Difficulties Questionnaire in Australia. Aust Psychol. 2005;40(3):215-222, Culpin et al (2015). Sleep problems and depression in adolescence: results from a large population-based study of Norwegian adolescents aged 16–18 years. European child & adolescent psychiatry, 23(8), 681-689

# Participant characteristics and missingness of study cohort compared with the cohort of participants who were invited to complete the ALSPAC Life@25+ Questionnaire and the full ALSPAC cohort

Table A2 displays participant characteristics of this study cohort, those who were invited to compete the ALSPAC Life@25+ Questionnaire and the whole ALSPAC cohort. There was a higher proportion of female participants (66.2%) in this study cohort than in the cohort of participants who were invited to complete the ALSPAC Life@25 Questionnaire (52.4%) and the whole ALSPAC cohort (48.9%). Educational attainment was slightly higher in this study cohort than in the whole cohort, mean scores were 13.9 vs 12.8 and 10.7 vs 9.2 on entry assessment and key stage 1 tests respectively.

Fewer participants in this study cohort had language difficulties than in the full cohort at age 9 years 7 months, 1.2% vs 1.6% children tended to leave out words and 7.4% vs 8.3% children sometimes made mistakes with pronouns. Over 98% of participants in the three cohorts were healthy or very healthy and had comparable average of 1.1 to 1.2 number of different conditions child saw doctor for in past year at age 6 years and 9 months. Sleeping patterns were similar across the three cohorts with sleeping difficulties more common at age 6 years and 9 months than 11 years and 8 months.

There was a lower percentage of participants with SDQ externalising construct score above the 90^th^ percentile at ages 6 years 9 months (7.2% vs 10%) and 11 years 8 months (8.8% vs 13.3%) in this study cohort compared to the full ALSPAC cohort. The mean SDQ internalising construct score was similar across cohorts and ages ranging 2.5 to 2.6, with the percentage above the 90^th^ percentile ranging 12.2% to 12.7% at age 6 years 9 months and between 12.5% and 13.4% at age 11 years 8 months.

The percentage of children with a depression score above the 90^th^ percentile was slightly lower for this study cohort compared to the whole cohort, 9.3% vs 10.5% and 10.7% vs 12.1% at 9 years 7 months and 11 years 8 months respectively.

There was a slightly higher percentage of participants in the most deprived geographic area in this study cohort compared to the whole cohort, 34.8% vs 30.8% and a lower percentage in the least deprived area 11.7% vs 14.9%. A smaller percentage of mothers had no education qualification (2.5% vs 4.3%), a higher percentage had higher education (32.4% vs 24.6%) and owned her house (86.6% vs 81%). Above 93% of mothers described their current health as well and healthy in the three cohorts and the prevalence of anxiety and depression was similar, with a smaller percentage of depression (21.1 vs 23.7) in this study cohort compared to the whole cohort.

The percentage of missingness across characteristics (Table A3) ranged from 0% to 35.5% in the cohort analysed, 0% to 37.8% in the cohort of participants who were invited to complete the ALSPAC Life@25+ Questionnaire and 0% to 52.5% in the whole ALSPAC cohort. Missingness percentages across characteristics were 10 points in average lower in this study cohort than those for the cohort of participants who were sent the ALSPAC Life@25+ Questionnaire, except for educational and environmental measures where differences in missingness rates ranged between 0.2 and 7.1 points.

**Table A2. Participant characteristics by cohort**

|  | **Completed HLS-EU-Q16 (N=4248)** | **invited to complete the ALSPAC Life@25+ Questionnaire (N= 9989)** | **Full ALSPAC cohort (N= 14878)** |
| --- | --- | --- | --- |
| **Sex, Female, N (%)** | 2812 (66.2) | 5235 (52.4) | 7342 (48.9) |
| **Entry assessment score, Mean (SD)** | 13.9 (3.1) | 13.2 (3.2) | 12.8 (3.2) |
| **Key Stage 1 score, Mean (SD)** | 10.7 (3.3) | 9.8 (3.6) | 9.2 (3.7) |
| **Key Stage 2 score, Mean (SD)** |  |  |  |
| English | 4.4 (0.7) | 4.2 (0.8) | 4.1 (0.9) |
| Mathematics | 4.3 (0.8) | 4.2 (0.8) | 4.1 (0.9) |
| Science | 4.6 (0.6) | 4.4 (0.7) | 4.4 (0.7) |
| **Child's speech is mostly just two or three word phrases (9y 7m), True, N (%)** | 8 (0.2) | 22 (0.3) | 44 (0.5) |
| **Child can produce long and complicated sentences (9y 7m), True, N (%)** | 3365 (98.6) | 6829 (98.5) | 7945 (98.2) |
| **Child tends to leave out words and grammatical endings (9y 7m), True, N (%)** | 40 (1.2) | 90 (1.3) | 130 (1.6) |
| **Child sometimes makes mistakes with pronouns (9y 7m), True, N (%)** | 253 (7.4) | 543 (7.9) | 666 (8.3) |
| **Assessment of child's health in past year, Very healthy or healthy, N (%)** |  |  |  |
| 6y 9m | 3067 (98.3) | 6321 (98.3) | 7619 (98.2) |
| 11y 8m | 3104 (98.2) | 6118 (98.5) | 6953 (98.5) |
| **No. different conditions child saw doctor for in past year (6y 9m), Mean (SD)** | 1.1 (1.7) | 1.2 (1.7) | 1.2 (1.7) |
| **Child had difficulty going to sleep in past year, Yes, N (%)** |  |  |  |
| 6y 9m | 2139 (64.7) | 4411 (63.6) | 5305 (63.0) |
| 9y 7m | 1790 (52.7) | 3620 (52.3) | 4233 (52.5) |
| **Child had nightmares in past year, Yes, N (%)** |  |  |  |
| 6y 9m | 1653 (50.3) | 3419 (49.6) | 4134 (49.5) |
| 9y 7m | 935 (28.0) | 1981 (29.1) | 2331 (29.4) |
| **Child got up after few hrs sleep in past year, Yes, N (%)** |  |  |  |
| 6y 9m | 353 (10.7) | 841 (12.2) | 1033 (12.3) |
| 9y 7m | 184 (5.4) | 427 (6.2) | 529 (6.6) |
| **SDQ externalising construct score, Mean (SD)** |  |  |  |
| 6y 9m | 4.5 (3.1) | 4.9 (3.3) | 5.0 (3.3) |
| 11y 8m | 3.5 (2.9) | 3.9 (3.1) | 4.0 (3.2) |
| **SDQ externalising construct, score above 90th percentile, N (%)** |  |  |  |
| 6y 9m | 236 (7.2) | 647 (9.4) | 839 (10.0) |
| 11y 8m | 291 (8.8) | 803 (12.4) | 970 (13.3) |
| **SDQ internalising construct score, Mean (SD)** |  |  |  |
| 6y 9m | 2.5 (2.5) | 2.5 (2.5) | 2.6 (2.5) |
| 11y 8m | 2.5 (2.7) | 2.6 (2.7) | 2.6 (2.7) |
| **SDQ internalising construct, score above 90th percentile, N (%)** |  |  |  |
| 6y 9m | 405 (12.2) | 864 (12.5) | 1068 (12.7) |
| 11y 8m | 412 (12.5) | 852 (13.2) | 980 (13.4) |
| **SMFQ depression score, Mean (SD)** |  |  |  |
| 9y 7m | 2.4 (3.0) | 2.5 (3.2) | 2.6 (3.3) |
| 11y 8m | 2.2 (3.1) | 2.3 (3.1) | 2.3 (3.2) |
| **SMFQ depression, score above 90th percentile, N (%)** |  |  |  |
| 9y 7m | 315 (9.3) | 690 (10.0) | 844 (10.5) |
| 11y 8m | 351 (10.7) | 754 (11.7) | 881 (12.1) |
| **2000 IMD score quintiles** |  |  |  |
| 1 | 1058 (34.8) | 2051 (32.2) | 2360 (30.8) |
| 2 | 608 (20.0) | 1255 (19.7) | 1518 (19.8) |
| 3 | 557 (18.3) | 1169 (18.3) | 1406 (18.3) |
| 4 | 465 (15.3) | 1002 (15.7) | 1237 (16.1) |
| 5 | 355 (11.7) | 896 (14.1) | 1143 (14.9) |
| **Mother's educational level** |  |  |  |
| No educational qualification | 83 (2.5) | 273 (3.8) | 377 (4.3) |
| O-levels, CSE/GCSE | 668 (20.1) | 1706 (24.0) | 2214 (25.1) |
| Further education | 1500 (45.1) | 3258 (45.8) | 4050 (46.0) |
| Higher education | 1077 (32.4) | 1882 (26.4) | 2172 (24.6) |
| **Mother's home ownership status, Owned, N (%)** | 2917 (86.6) | 5973 (82.5) | 7287 (81.0) |
| **Mother's description of current health, Well and healthy, N (%)** | 3128 (93.8) | 6563 (93.8) | 8005 (93.3) |
| **Mother has had anxiety or ‘nerves’ in last 3 years, Yes, N (%)** | 698 (21.0) | 1505 (21.6) | 1919 (22.5) |
| **Mother has had depression in last 3 years, Yes, N (%)** | 701 (21.1) | 1594 (22.8) | 2026 (23.7) |

HLS-EU-Q16: Health Literacy Survey Questionnaire- short version; SD: Standard deviation; SDQ: Strengths and Difficulties Questionnaire; SMFQ: Short Mood and Feelings Questionnaire; IMD: Index of Multiple Deprivation; CSE: A qualification in a specific subject formerly taken by school students aged 14–16; it was the lowest of three levels of standardised British examinations in a secondary school subject, at a level below O-level. CSE and O-level were replaced in 1988 by the GCSE; GCSE is a qualification in a specific subject typically taken by school students aged 14–16

**Table A3. Percentage of missingness by cohort**

| **Variable** | **Completed HLS-EU-Q16 (N=4248)** | | **Invited to complete ALSPAC Life@25 Questionnaire (N= 9989)** | **Full ALSPAC sample (N= 14878)** |
| --- | --- | --- | --- | --- |
| Child's sex | | 0.0 | 0.0 | 0.0 |
| Entry assessment score | | 35.5 | 34.3 | 40.9 |
| Key Stage 1 score | | 22.4 | 22.1 | 30.1 |
| Key Stage 2 English score | | 13.6 | 15.7 | 26.1 |
| Key Stage 2 Mathematics score | | 14.2 | 15.9 | 26.3 |
| Key Stage 2 Science score | | 13.5 | 14.8 | 24.9 |
| Child's speech is mostly just two or three word phrases (9y 7m) | | 19.9 | 30.6 | 45.6 |
| Child can produce long and complicated sentences (9y 7m) | | 19.7 | 30.6 | 45.5 |
| Child tends to leave out words and grammatical endings (9y 7m) | | 19.8 | 30.6 | 45.6 |
| Child sometimes makes mistakes with pronouns (9y 7m) | | 20.0 | 30.8 | 45.7 |
| Assessment of child's health in past year (6y 9m) | | 26.5 | 35.6 | 47.8 |
| Assessment of child's health in past year (11y 8m) | | 25.6 | 37.8 | 52.5 |
| No. different conditions child saw doctor for in past year (6y 9m) | | 21.6 | 30.1 | 42.9 |
| Child had difficulty going to sleep in past year (6y 9m) | | 22.2 | 30.5 | 43.3 |
| Child had difficulty going to sleep in past year (9y 7m) | | 20.0 | 30.7 | 45.7 |
| Child had nightmares in past year (6y 9m) | | 22.6 | 31.0 | 43.8 |
| Child had nightmares in past year (9y 7m) | | 21.3 | 31.8 | 46.6 |
| Child got up after few hrs sleep in past year (6y 9m) | | 22.4 | 30.8 | 43.5 |
| Child got up after only few hours sleep in past year (9y 7m) | | 20.3 | 31.0 | 45.9 |
| SDQ externalising construct score (6y 9m) | | 22.4 | 30.9 | 43.7 |
| SDQ externalising construct score (11y 8m) | | 22.5 | 35.3 | 50.8 |
| SDQ internalising construct score (6y 9m) | | 22.1 | 30.7 | 43.5 |
| SDQ internalising construct score (11y 8m) | | 22.4 | 35.3 | 50.8 |
| SMFQ depression score (9y 7m) | | 20.1 | 30.8 | 45.8 |
| SMFQ depression score (11y 8m) | | 22.7 | 35.5 | 51.0 |
| 2000 IMD score quintiles | | 28.4 | 36.2 | 48.4 |
| Mother's educational level | | 21.7 | 28.7 | 40.7 |
| Mother's home ownership status | | 20.7 | 27.6 | 39.5 |
| Mother's description of current health | | 21.5 | 29.9 | 42.3 |
| Mother has had anxiety or 'nerves' in last 3 years | | 21.7 | 30.1 | 42.5 |
| Mother has had depression in last 3 years | | 21.7 | 30.2 | 42.5 |

HLS-EU-Q16: Health Literacy Survey Questionnaire- short version; SD: Standard deviation; SDQ: Strengths and Difficulties Questionnaire; SMFQ: Short Mood and Feelings Questionnaire; IMD: Index of Multiple Deprivation; CSE: A qualification in a specific subject formerly taken by school students aged 14–16; it was the lowest of three levels of standardised British examinations in a secondary school subject, at a level below O-level. CSE and O-level were replaced in 1988 by the GCSE; GCSE is a qualification in a specific subject typically taken by school students aged 14–16
